# Protocol for a prospective randomized controlled trial of recipient remote ischaemic preconditioning in orthotopic liver transplantation (RIPCOLT trial)

**DOI:** 10.1186/s13737-016-0033-4

**Published:** 2016-04-06

**Authors:** Francis P. Robertson, Rup Goswami, Graham P. Wright, Barry Fuller, Brian R. Davidson

**Affiliations:** Department of Surgery and Interventional Science, Royal Free Campus, Royal Free Hospital, University College London, 9th Floor, Pond Street, London, NW3 2QG UK; Department of Immunology, Napier University, Edinburgh, UK; HPB and Liver Transplant Unit, Royal Free London NHS Foundation Trust, Pond Street, London, NW3 2QG UK

**Keywords:** Liver transplantation, Remote ischaemic preconditioning, Ischaemia reperfusion injury, Outcome, Aspartate transferase

## Abstract

**Abstract:**

Ischaemic reperfusion (IR) injury is a major cause of graft loss, morbidity and mortality following orthotopic liver transplantation (OLT). Demand for liver transplantation has resulted in increasing use of marginal grafts that are more prone to IR injury. Remote ischaemic preconditioning (RIPC) reduces IR injury in experimental models, but recipient RIPC has not been evaluated clinically.

**Methods:**

A single-centre double-blind randomized controlled trial (RCT) is planned to test the hypothesis that recipient RIPC will reduce IR injury. RIPC will be performed following recipient anaesthetic induction but prior to skin incision. The protocol involves 3 cycles of 5 min of lower limb occlusion with a pneumatic tourniquet inflated to 200 mmHg alternating with 5 min of reperfusion. In the control group, the sham will involve the cuff being placed on the thigh but without being inflated.

The primary endpoint is ability to recruit patients to the trial and safety of RIPC. The key secondary endpoint is a reduction in serum aspartate transferase levels on the third post-operative day.

**Discussion:**

RIPC is a promising strategy to reduce IR injury in liver transplant recipients as there is a clear experimental basis, and the intervention is both inexpensive and easy to perform. This is the first trial to investigate RIPC in liver transplant recipients.

**Trial registration:**

Clinicaltrials.gov NCT00796588

**Electronic supplementary material:**

The online version of this article (doi:10.1186/s13737-016-0033-4) contains supplementary material, which is available to authorized users.

Ischaemia reperfusion (IR) injury, the injury which occurs when an organ’s blood supply is interrupted and reconstituted, is a major cause of morbidity and mortality post liver transplantation and is believed to account for at least 10 % of early graft loss [1]. Grafts from extended criteria donors are more prone to IR injury and the use of grafts from donors after cardiac death (DCD) is associated with a two-fold increase in risk of death and graft loss post transplantation in UK centres [2]. Due to a recent widening of the criteria for liver transplantation, there is greater demand for an already scare resource. In the UK, the use of grafts from DCD donors has increased from 6.9 % in 2005 [2] to 19.1 % of grafts implanted in 2013 [3]. There is no current accepted treatment of IR injury and as such strategies to ameliorate IR injury are of key clinical importance.

Ischaemic preconditioning (IPC), first described in 1986 in a canine cardiac model [4] has been shown to ameliorate warm hepatic IR injury in small animal models. Several small trials have been performed investigating the role of IPC of donor livers prior to retrieval [5–8]. The results are conflicting with only one trial demonstrating a better clinical outcome with fewer clinical episodes of clinical rejection in grafts from extended criteria donors following IPC [7], and two trials demonstrating a reduction in serum transaminases post transplantation [7, 8] with one trial reporting higher transaminase levels post transplantation following IPC [5]. No trial was able to demonstrate a reduction in graft loss or post-operative morbidity and mortality; however, none of the trials were powered to demonstrate a reduction in morbidity or mortality.

Paradoxically in small animal models, IPC has been shown to place added stress on the recovering liver impairing liver regeneration [9, 10], and in a multi-variate analysis of patients undergoing hepatic resection, IPC was found to be an independent risk factor for post-operative morbidity [11].

Remote ischaemic preconditioning (RIPC), first described in 1993 again in a canine cardiac model [12] has been found to provide protection against IR injury without adding stress to the target organ and as such is an attractive prospect for reducing IR injury in liver transplant recipients. Although RIPC has been shown to ameliorate hepatic IR injury in small animal models [13, 14], there has been no human liver transplant trial. Three recent trials have been performed in kidney transplantation including both living [15, 16] and deceased donors [17]. Two trials demonstrated evidence of an improvement in early graft function [15, 17]; however, one trial failed to demonstrate any evidence of improved graft function or reduced levels of biomarkers of graft injury [16]. Our aim is to perform a double-blinded randomized control trial to investigate the protection gained by RIPC in liver transplant recipients.

## Methods/design

### Study design and setting

RIPCOLT has been designed as a prospective double-blind randomized control trial with both surgeon and patient blinded to whether the recipient has received preconditioning. This protocol has been designed according to the SPIRIT guidelines [18]. The pilot will be carried out in a single centre—the Royal Free Hospital, London, with a subsequent multi-centre trial to establish cost effectiveness.

### Ethical approval

This trial has been approved by both the ethical board of the National Research Ethical Service (11/H0720/4) and the Royal Free Hospital/University College London ethical board (8191).

The trial has been registered with clinicaltrials.gov (NCT00796588).

### Participants

The pilot study will include 50 patients undergoing elective deceased donor liver transplantation at the Royal Free Hospital, London. Data will then be analysed regarding trial recruitment, completion of RIPC protocol, safety and preliminary evidence of efficacy to allow the endpoints and power of a subsequent multi-centre RCT to be designed. All patients undergoing deceased donor liver transplantation at the Royal Free Hospital will be considered for inclusion in the trial; exclusion criteria are contained in Table 1.

**Table 1:** Exclusion criteria for trial

Exclusion criteria	Re-transplantation
	Patients under 16 years of age
	Super-urgent transplantation
	Lack of informed consent
	Combined liver and kidney transplantation
	Peripheral vascular disease
	Varicose veins
	Localized limb infection
	Prior history of thrombo-embolic disease
	Inclusion in another interventional trial

### Recruitment

Patients will be identified upon admission for transplant assessment and will be screened for inclusion and exclusion criteria. They will be provided with a patient information sheet and allowed 24 h to review the information prior to consenting by a member of the research team. A copy of the consent form and patient information sheet is included as an Additional file 1.

### Power calculation

A power calculation is not required for a pilot feasibility study [19]. However, guidance would suggest that 50 patients would be suitable for a feasibility study [20] with 25 randomized to receive RIPC and 25 to liver transplantation as per standard.

### Randomization

Participants are randomized to either the control or intervention group following induction of anaesthesia and collection of baseline blood samples. Randomization is performed via a random number sealed envelope system. Both the surgeon and the patient will be blinded to whether the patient receives RIPC.

### Trial protocol

Induction and maintenance of anaesthesia will be performed by intravenous propofol. The use of volatile inhaled induction agents such as sevoflurane have been shown to exert a degree of pharmacological preconditioning [21, 22] and as such they will be avoided.

Following induction but before the intervention, baseline blood and urine samples will be collected for measurement of pre-operative circulating cytokine levels and markers of renal parenchymal injury in both the blood and the urine.

A layer of stockinette will be applied to the left upper thigh with a wide pneumatic tourniquet applied over it in accordance with safe and recommended practices by the Association of Peri-operative Registered Nurses (AORN) [23] and Royal Free Hospital (RFH) departmental guidelines.

In those undergoing RIPC, the tourniquet will be inflated to 200 mmHg for 5 min followed by 5 min of reperfusion. This will be repeated three times.

In the control group, a sham will consist of the pneumatic tourniquet being placed on the left upper thigh without being inflated. The transplant will then proceed as standard.

Further blood samples will be collected 2 h following reperfusion of the graft to measure circulating cytokine levels post reperfusion. At the same time, two post reperfusion biopsies will be obtained. One of these biopsies will be fixed in 10 % natural buffered formalin for 48 h before being embedded in paraffin for H&E staining. IR injury will be graded by a pathologist according to the Suzuki classification of liver IR injury [24]. The second biopsy will be processed to extract fresh intra-hepatic lymphocytes for analysis by flow cytometry.

A further blood and urine sample will be obtained 24 h post reperfusion to measure circulating cytokine levels and markers of renal parenchymal injury both in the blood and urine.

All patients will receive standard post-operative care.

**Fig. 1:**
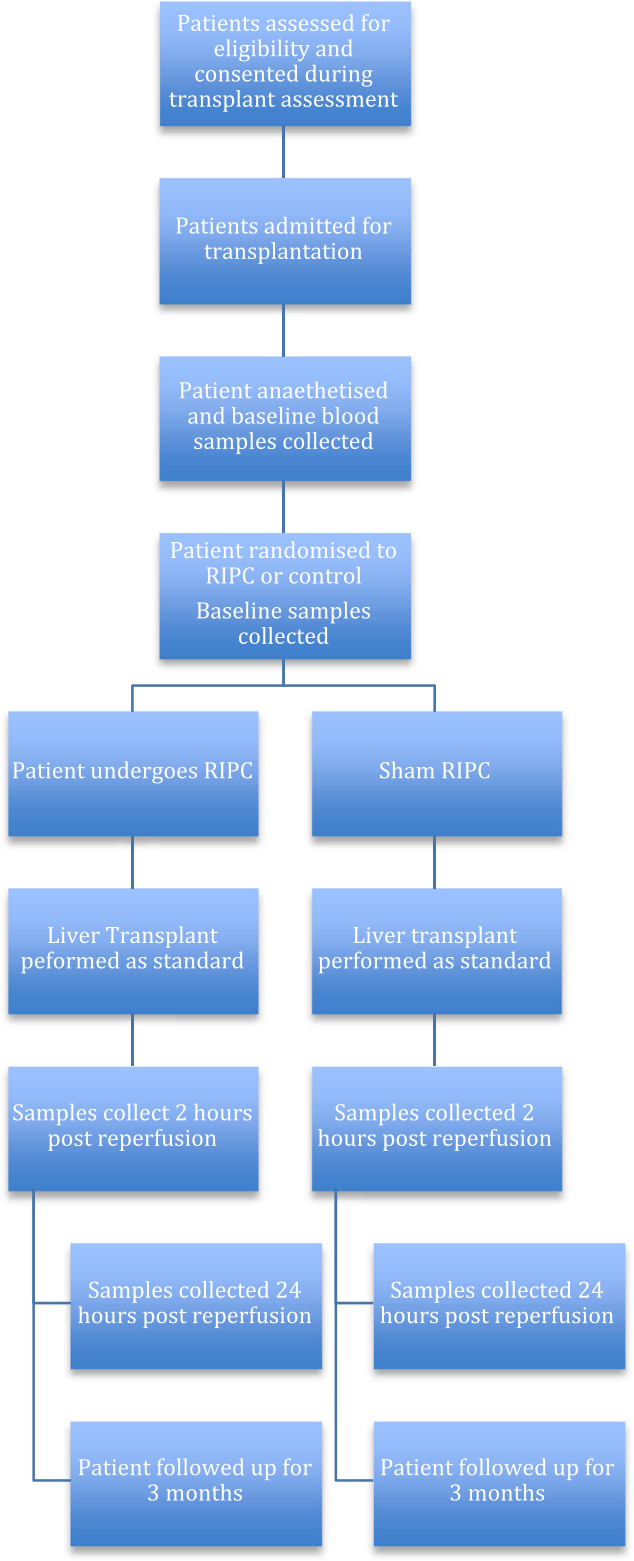
Trial flow chart

Routine clinical data will be collected including local complications as a result of RIPC, liver biochemistry, clotting profiles, urea and creatinine levels, post-operative complications, requirements for organ support, length of stay in the intensive care department and total hospital stay. Patients will be followed up for 3 months. A flow diagram of the trial is included in Fig. 1.

### Outcome measures

The aim of this study is to assess the feasibility of recruiting liver transplant recipients and performing RIPC immediately prior to liver transplantations. The primary and secondary outcomes (Table 2) will help design a subsequent multi-centre RCT of RIPC in liver transplant recipients.

**Table 2:** Primary and secondary endpoints

Primary endpoints	Ability to recruit patients to the trial
	Feasibility of performing RIPC in liver transplant recipients
	Safety of RIPC in liver transplant recipients
Secondary endpoints	AST levels on the third post-operative day [30]
	Incidence of acute kidney injury and need for renal replacement therapy
	Length of stay in intensive care and total hospital stay
	Incidence of vascular thrombotic events
	Incidence of biliary complications
	Incidence of post-operative infections
	Incidence of acute rejection in the first months post transplantation
	Circulating cytokine levels 2 h post reperfusion of the liver graft
	T cell accumulation and activation in the liver 2 h post reperfusion
	Urinary and serum NGAL levels 2 h post reperfusion

The criteria to progress to a full trial will be determined based on a consent rate of at least 60 % and post randomization dropout rate from the study of less than 30 %.

The safety of RIPC will be measured by incidence of localized complications following RIPC to include the development of a deep vein thrombosis or pulmonary embolus, the development of localized cellulitis and pain or paresthesia of the left lower limb following RIPC.

### Data analysis

Data will be stored on password-protected UCL computers in locked departments and will be anonymised prior to use. Since this is a feasibility study, all analyses other than recruitment rate and dropout rates should be considered exploratory. RIPC and control groups will be compared including both baseline characteristics and secondary outcomes. Continuous variables will be presented using means and standard deviations or medians and inter-quartile ranges, as appropriate, and binary variables will be presented as frequency counts and percentages. The mean difference in proportions (for binary outcomes) and the mean or median difference (for continuous outcomes) between the two groups will be explored and presented with 95 % confidence intervals.

This important data will be used to help the design of the multi-centre RCT.

Results will be analysed via Statistical Package for Social Sciences (SPSS) (IBM Chicago, IL, USA).

### Dissemination

It is anticipated that the results of the study will be presented at the British Transplant Society annual meeting and reported in a peer-reviewed journal of interest to the transplant community.

We will publish a research report on the University College London’s website and the Royal Free NHS trusts’ website.

## Discussion

IR injury is a major cause of graft loss, morbidity and mortality following liver transplantation [1, 25]. Due to the increase utilization of grafts from extended criteria donors which are more prone to IR injury [2], strategies to ameliorate IR injury are a key research goal. We have previously shown that RIPC reduces hepatic IR injury in small animal models [13, 14, 26]. Previous trials have investigated IPC of donor livers prior to retrieval [5–8] but failed to discern any clinical benefit. IR injury is not confined to the liver but results in a systemic cytokine storm, immune activation and multi-organ dysfunction. In successful small animal models, preconditioning is performed in the same individual as the reperfusion injury occurs and therefore the process of performing RIPC in the recipient may be more efficacious at reducing the systemic inflammatory response associated with IR injury reducing graft injury and end organ damage.

Several different protocols for RIPC exist, and there is no current consensus as to which protocol is best. Three cycles of 5 min of ischaemia have been shown to ameliorate IR injury in small animal models [27] and in patients undergoing coronary artery bypass graft surgery [28], and as such we have elected to use this preconditioning protocol in this trial.

This is the first trial to investigate whether recipient RIPC can improve the outcome of human liver transplant recipients. As such, we have chosen to perform a pilot feasibility study of 50 patients initially. We have designed this pilot study as a blinded randomized control trial to give us the strongest pilot data to adequately power and design the main trial. The data from this initial patient cohort will allow us to determine patient recruitment, support of the liver transplant physicians and surgeons, the feasibility of carrying out the period of preconditioning between anaesthesia and commencing surgery, the safety of limb ischaemia in liver transplant recipients and to gain preliminary data on efficacy with which to determine the endpoints and power required for a subsequent multi-centre RCT.

We have chosen to exclude patients with a history of venous thrombo-embolic disease, peripheral arterial disease and localized limb infections to minimize risk to participating patients during a yet unproven intervention. We have also chosen to exclude patients undergoing liver transplantation for acute liver failure, on the super-urgent list, as a transplantation in this setting is associated with a threefold increased risk of morbidity and mortality [29]. In a small trial, this would introduce significant bias to the results. Furthermore, these patients have altered levels of consciousness due to encephalopathy and consent is a problem for their inclusion in this feasibility study. Patients undergoing simultaneous liver kidney transplantation have been excluded to remove the bias of the added surgical stress and the inclusion of patients with severe pre-operative renal impairment who are offered combined transplants.

We have chosen AST on the third post-operative day as an important endpoint as recent work has shown that this correlates strongly with overall graft function, post-operative mortality, need for organ support and incidence of post-operative infective complications [30]. With current 90-day graft loss rates of 3.5 % and 90-day mortality rates of 6.9 % following elective liver transplant in the UK [29], a very large RCT would be required to determine significant benefit following RIPC based on these endpoints which is currently unfeasible. We have also chosen to measure urinary and serum neutrophil gelatinase-associated lipocalin (NGAL) levels as a sensitive marker [31] of evidence of acute kidney injury (AKI) post transplantation as this is a common complication post liver transplantation, and reduction in the incidence of end organ damage would provide a further hard endpoint to be used as an outcome in the future RCT in combination with day 3 AST levels.

Evidence of a reduction in IR injury and the associated systemic inflammatory response will be measured by analysing the extent of IR injury and immune cell activation in post reperfusion biopsies and circulating serum cytokines.

In summary, this is the first trial to investigate RIPC in the setting of liver transplantation. RIPC has been shown to ameliorate IR injury in renal transplantation [15, 17]. Samples from this trial will be analysed to provide evidence of a reduction in immune activation by RIPC.

If successful, this inexpensive holistic intervention could improve outcome following liver transplantation and allow the implantation of more grafts from extended criteria donors—widening the donor pool.

## Additional file

Additional file 1: Patient Information Sheet & Consent Form. (DOC 43 kb)

## Abbreviations

AKI: acute kidney injury
AORN: Association of Peri-operative Registered Nurses
AST: aspartate transferase
DCD: donor after cardiac death
IPC: ischaemic preconditioning
IR: ischaemia reperfusion
mmHg: millimetres of mercury
OLT: orthotopic liver transplantation
RCT: randomized controlled trial
RFH: Royal Free Hospital
RIP: remote ischaemic preconditioning
