# Supplementary material for: Protocol for a prospective randomized controlled trial of recipient remote ischaemic preconditioning in orthotopic liver transplantation (RIPCOLT trial)
Source: Transplant Res. 2016 Apr 6;5:4. doi: 10.1186/s13737-016-0033-4 (PMC4822296; doi:10.1186/s13737-016-0033-4)
Supplement: Additional file 1: — Patient Information Sheet & Consent Form. (DOC 43 kb) [file 13737_2016_33_MOESM1_ESM.doc]

**PATIENT INFORMATION SHEET & CONSENT FORM**

**Study title:** Remote Ischaemic Preconditioning and Outcome of Liver Transplantation.

**Invitation**

You are being invited to take part in a research study. Before you decide it is important for you to understand why the research is being done and what it will involve. Please take time to read the following information carefully and discuss it with friends and relatives if you wish. Ask us if there is anything that is not clear or if you would like more information. Take time to decide whether or not you wish to take part.

Thank you for reading this.

1. **What is the purpose of the study?**

About 650 liver transplants are performed each year in the UK. During the transport period of the organs between the donor centre and recipient centre, the organs are damaged by the absence of blood flow (ischaemia) and when they are transplanted and blood flow is restored there is further damage (reperfusion injury). Brief reduction of blood flow to the arm or leg has been shown to reduce the effects of ischaemia and reperfusion injury (remote ischaemic preconditioning, RIPC) encountered after many types of surgery but has not been applied to liver transplantation. This study is aimed at determining whether RIPC is effective in decreasing ischaemia-reperfusion injury (I/R injury) in patients undergoing liver transplantation.

1. **Why have I been chosen?**

All patients waiting to undergo liver transplantation will be invited to take part in the study. Once entered into the study you will be randomly allocated to RIPC group or no RIPC (control or standard treatment) group.

1. **Can I refuse to take part in the study or withdraw from the study?**

You do not have to take part in the study or you can withdraw at any stage. This will not affect your treatment in any way. If you refuse to participate in the study or choose to withdraw from the study before the intervention (RIPC or standard treatment) is carried out, we will provide you with the standard treatment. No further data will be collected. If you choose to withdraw from the study after the intervention is carried out, we will use the data already collected while presenting the results, but will not collect any further data from the time of your withdrawal.

1. **What will happen to me if I take part?**

- While you are undergoing anaesthesia (being put to sleep for the purposes of the transplantation), a blood pressure cuff will be inflated around one of your legs for 5 minutes. This will then be deflated for a period of 5 minutes and then inflated again for a period of 5 minutes. This inflation and deflation cycles will be carried out thrice. The inflation pressure will not cause any damage to the legs or to the circulation, as frequently, bone doctors inflate the cuff for a continuous period of more than 1 hour while performing some operations. We perform this only for a 5 minute continuous period. Since you will be asleep during the procedure you will not feel any discomfort from the intervention. There will no increase in the time of the anaesthesia since this procedure will be carried out while other preparations for the surgery are performed. Apart from this inflation and deflation of cuff, there will be no difference in the care that people who participate and do not participate receive.
- As part of routine care, a bit of liver tissue (biopsy) is taken from you after liver transplantation. We will use some of that tissue for research purposes.
- A second biopsy will be taken at the same time, which will be used for research purposes.
- We will measure pressure in the portal vein (the vein carrying blood to the liver) at the end of liver transplant procedure.
- Similarly, blood is routinely taken at the time of transplantation and after transplantation. The results of these blood tests will be collected as part of this research. In addition, data routinely collected by the NHS Blood and Transplant (NHSBT) to monitor your progress will also be used in this research.
- We would measure markers of ischaemia-reperfusion injury in the blood samples collected routinely.
- Similarly, we will measure markers of ischaemia-reperfusion injury in the urine.
- Information will also be sought from routine biopsies in the post-operative period which is done to exclude suspected rejection.

1. **Will there be any changes to my treatment because of this research?**

Apart from this inflation and deflation of the blood pressure cuff, there will be no difference in the care that people who participate and do not participate receive.

1. **What do I have to do?**

There are no changes to your routine treatment or restrictions imposed on you from taking part in this research.

1. **What are the side effects of taking part?**

We anticipate no disadvantages of having the cuff applied. In a few patients where the tourniquet has been applied improperly or for more than half hour continuously, side effects have included pain in the leg, tingling and numbness and rarely damage to the blood vessel of the leg. However, we are applying the cuff for a continuous period of 5 minutes only.

1. **What are the possible disadvantages and risks of taking part?**

We do not anticipate any disadvantage or significant risks from taking part in this trial. Although this type of research has not been conducted in liver transplantation patients, cycles of brief inflation and deflation of the cuff has been performed in patients undergoing removal of part of liver, in patients donating their kidneys for their relatives, and in patients undergoing kidney transplantation with no reported disadvantages or risks.

1. **What are the possible benefits of taking part?**

RIPC may reduce the damage to your liver. The information we get from this study may help us to understand why livers get damaged during liver surgery and whether a simple interventional procedure will prevent this damage.

1. **Will my GP be informed of this research?**

We will not inform your GP routinely that you have participated in this research, as there will be no difference in the care that people who participate and do not participate receive, apart from this inflation and deflation of the blood pressure cuff.

1. **What if something goes wrong?**

Compression cuff is used routinely for some surgical procedures on the legs and has been shown to be safe and effective. If there was any complication as a result of this research, you would be treated under the NHS and will be covered under the NHS indemnity insurance.

1. **Will my taking part in this study be kept confidential?**

All information which is collected about you during the course of the research will be kept strictly confidential. Any information about you will stored using hospital numbers rather than using identifiable information names, address, and date of birth.

1. **What will happen to the results of the research study?**

The results from the study may be published in medical journals anytime between 6 to 12 months following completion of the study, but you will not be identified.

1. **Who is organising and funding the research?**

The study is being supported by the Department of Hepatology at the Royal Free Hospital. There are no additional payments either to the doctor or patients for being involved in the study.

1. **Who has reviewed the study?**

The study has been reviewed and approved by the Research Ethics Committee of the Royal Free Hospital.

1. **Contact for Further Information**

Prof Brian Davidson, MD, FRCS,
Professor of HPB and Liver Transplantation Surgery
9^th^ Floor, Royal Free Hospital,
Pond Street,
London.
NW3 2QG
Telephone: 0207 794 0500

We thank you for reading this information sheet. Please keep a copy of this information sheet and the signed consent form for your records if you agree to participate in this study.

**CONSENT FORM**

**Title of Project:**  Remote Ischaemic Preconditioning and Outcome of Liver Transplantation.

Ethics approval number:

Hospital number of the patient:

1. I confirm that I have read and understand the information sheet dated 19^th^ July 2011 (version 3) for the above study and have had the opportunity to ask questions.

2. I understand that my participation is voluntary and that I am free to withdraw at any time, without giving any reason, without my medical care or legal rights being affected.

3. I understand that sections of any of my medical notes may be looked at by responsible individuals or from regulatory authorities where it is relevant to my taking part in research. I give permission for these individuals to have access to my records.

4. I agree to take part in the above study.

________________________ ________________ ________________

Name of patient Date Signature

_________________________ ________________ _________________

Name of person taking consent Date Signature

_________________________ ________________ _________________

Researcher Date Signature
